# Collective near-field coupling and nonlocal phenomena in infrared-phononic metasurfaces for nano-light canalization

**DOI:** 10.1038/s41467-020-17425-9

**Published:** 2020-07-21

**Authors:** Peining Li, Guangwei Hu, Irene Dolado, Mykhailo Tymchenko, Cheng-Wei Qiu, Francisco Javier Alfaro-Mozaz, Fèlix Casanova, Luis E. Hueso, Song Liu, James H. Edgar, Saül Vélez, Andrea Alu, Rainer Hillenbrand

**Affiliations:** 1 Wuhan National Laboratory for Optoelectronics & School of Optical and Electronic Information, Huazhong University of Science and Technology, Wuhan, 430074 China; 2 CIC nanoGUNE BRTA, 20018 Donostia-San Sebastián, Spain; 3 Department of Electrical and Computer Engineering, National University of Singapore, 4 Engineering Drive 3, Singapore, 117583 Singapore; 4 Advanced Science Research Center, City University of New York, New York, NY 10031 USA; 5 IKERBASQUE, Basque Foundation for Science, 48013 Bilbao, Spain; 6 Tim Taylor Department of Chemical Engineering, Kansas State University, Manhattan, KS 66506 USA; 7 Department of Materials, ETH Zürich, 8093 Zürich, Switzerland; 8 Physics Program, Graduate Center, City University of New York, New York, NY 10016 USA; 9 CIC nanoGUNE BRTA and Department of Electricity and Electronics, UPV/ EHU, 20018 Donostia-San Sebastián, Spain

**Keywords:** Metamaterials, Nanophotonics and plasmonics, Polaritons

## Abstract

Polaritons – coupled excitations of photons and dipolar matter excitations – can propagate along anisotropic metasurfaces with either hyperbolic or elliptical dispersion. At the transition from hyperbolic to elliptical dispersion (corresponding to a topological transition), various intriguing phenomena are found, such as an enhancement of the photonic density of states, polariton canalization and hyperlensing. Here, we investigate theoretically and experimentally the topological transition, the polaritonic coupling and the strong nonlocal response in a uniaxial infrared-phononic metasurface, a grating of hexagonal boron nitride (hBN) nanoribbons. By hyperspectral infrared nanoimaging, we observe a synthetic transverse optical phonon resonance (strong collective near-field coupling of the nanoribbons) in the middle of the hBN Reststrahlen band, yielding a topological transition from hyperbolic to elliptical dispersion. We further visualize and characterize the spatial evolution of a deeply subwavelength canalization mode near the transition frequency, which is a collimated polariton that is the basis for hyperlensing and diffraction-less propagation.

## Abstract (web summary)

Phenomena such as polariton canalization and hyperlensing can be found at the transition from hyperbolic to elliptical dispersion. Here, the authors investigate this transition using hyperspectral infrared nanoimaging of polaritons in a grating of hexagonal boron nitride nanoribbons.

## Introduction

Uniaxial metasurfaces are thin layers of engineered subwavelength structures, whose in-plane effective permittivity tensor components are unequal (*ε*_eff,*x*_ ≠ *ε*_eff,*y*_), and thus support different types of in-plane anisotropic polaritons^1–10^. When both *ε*_eff,*x*_ and *ε*_eff,*y*_ are negative but with different absolute values, the polaritons propagating along the metasurface exhibit an elliptically-shaped dispersion diagram, i.e., the polariton momentum **k** describes an elliptical isofrequency contour (IFC) in *k*-space^6,9,11^. On the other hand, when *ε*_eff,*x*_ and *ε*_eff,*y*_ have opposite signs, polaritons possess a so-called hyperbolic dispersion (**k** describes hyperbolic IFCs in *k*-space)^1,4–9,12,13^, exhibiting increased polariton confinement and ray-like anisotropic propagation along the surface. These two types of anisotropic metasurfaces can be applied, for example, to enhance optical birefringence^14,15^, to control light polarization^16^, for nanoscale directional polariton guiding^5,8,9,17,18^, and for subwavelength-scale optical imaging^9,19^.

A particularly interesting regime arises when uniaxial metasurfaces exhibit a topological transition of the IFCs upon frequency variation^8,9,20^, changing from hyperbolic to elliptical. It offers unique opportunities in nanophotonics, for example, for enhancing the local photonic density of states^8,9,20^ and for supporting deeply subwavelength canalization modes^17,21^. These canalization modes can exhibit extremely anisotropic in-plane polariton momenta, which results in nanoscale and nearly diffraction-free electromagnetic energy transport with applications in hyperlensing^8,9,17^ and control of near-field heat transfer^9^. It has been shown theoretically that the topological transition and the canalization modes are determined by the polaritonic near-field coupling of subwavelength elements comprising the metasurfaces^7,9,17^. However, so far, the polaritonic coupling governing the topological transition has not been experimentally demonstrated. Only few experimental studies at microwave frequencies have visualized weakly confined canalization modes at 10 GHz^12^. Here, we demonstrate that the strong collective near-field coupling of subwavelength elements in an infrared-phononic metasurface (a hBN nanograting) yields a synthetic optical phonon resonance and subsequently a topological transition. By hyperspectral nanoimaging, we are able to observe the topological transition and the strong coupling of the nanoribbons (the metasurface elements in our case) in spatial and spectral domains. We also provide real-space images of deeply subwavelength canalization polaritons, which experimentally demonstrate that these modes are the consequence of the strong collective polaritonic near-field coupling of the nanoribbons and the associated strong nonlocal response of the metasurface.

## Results

### Nonlocal effective medium theory for a hBN metasurface

**Fig. 1:**
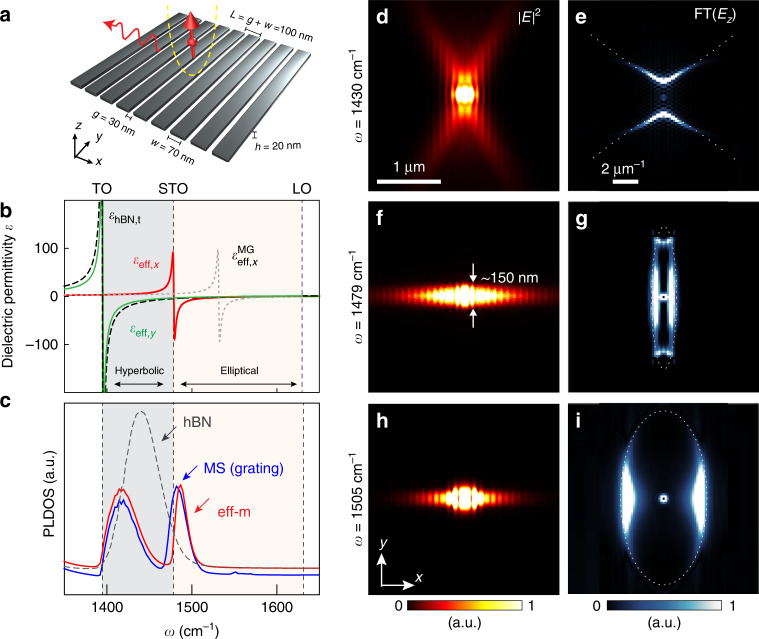
Topological transition and canalization of polaritons in a hBN metasurface. **a** Schematic of a 20-nm-thick hBN metasurface based on a grating made of nanoribbons. **b** Anisotropic effective dielectric permittivities (real parts) of the metasurface calculated using Eqs. 1–3, *ε*_eff,*x*_ (red line) and *ε*_eff,*y*_ (green). Permittivity of unpatterned hBN (*ε*_hBN,*t*_, dashed black line) and effective permittivity of the metasurface based on Maxwell–Garnett approximation ($\varepsilon^{\mathrm{MG}}_{{\mathrm{eff}},x}$, dashed gray line) are provided for comparison. **c** Simulated PLDOS on the grating metasurface (MS), the effective medium (eff-m) and the unpatterned hBN, respectively. **d**, **f**, **h** Near-field intensity distribution of dipole-launched polaritons at three different frequencies. **e**, **g**, **i** Absolute value of the Fourier transform (FT) of the simulated near-field distribution *E*_*z*_ (see Supplementary Fig. 4). Dotted hyperbola and ellipses are guides to the eye.

Boron nitride exhibits a negative and isotropic in-plane permittivity (*ε*_hBN,*t*_ = *ε*_*x*_ = *ε*_*y*_ < 0) and a positive out-of-plane permittivity *ε*_hBN,*z*_ in its upper mid-infrared Reststrahlen band (the frequency region between transversal and longitudinal optical phonon frequencies, TO and LO, respectively), where phonon polaritons (PhPs) exist^5,22–30^. Patterning a thin hBN flake into a periodic array of nanoribbons (nanograting) creates an infrared metasurface with strong in-plane anisotropy, which can support in-plane hyperbolic phonon polaritons when the near-field coupling between the ribbons is weak^5^ (Fig. 1a, d, e, ribbon width *w* = 70 nm, gap size *g* = 30 nm, thickness *h* = 20 nm). By considering now strong polaritonic near-field coupling of the ribbons and the subsequent strong nonlocal effect induced by the periodic structuring (the effective permittivity depends on the polariton momentum *k* that is controlled by the grating period *L*, see discussions in ref. ^8,9^), the effective anisotropic permittivity (*ε*_eff,*x*_, *ε*_eff,*y*,_ *ε*_eff,*z*_) of this metasurface needs to be described by a modified effective medium model^8,9^, yielding

1 $$\varepsilon _{{\mathrm{eff}},x} = \left( {\frac{{1 - \xi }}{{\varepsilon _{{\mathrm{hBN}},{\it{t}}}}} + \frac{\xi }{{\varepsilon _{\mathrm{c}}}}} \right)^{ - 1}$$

2 $$\varepsilon _{{\mathrm{eff}},y} = \left( {1 - \xi } \right)\varepsilon _{{\mathrm{hBN}},{\it{t}}} + \xi \varepsilon _{{\mathrm{air}}}$$

3 $$\varepsilon _{{\mathrm{eff}},z} = \left( {1 - \xi } \right)\varepsilon _{{\mathrm{hBN}},{\it{z}}} + \xi \varepsilon _{{\mathrm{air}}}$$

where $\xi = \frac{g}{L}$ is the filling factor. Importantly, *ε*_c_ is a nonlocal correction parameter capturing the polaritonic near-field coupling of adjacent ribbons and the corresponding nonlocal correction^8,9^, which depend on grating period, thickness, and filling factor.

We note that strong polaritonic near-field coupling of the hBN ribbons and the associated nonlocal response cannot be captured by a standard effective medium model based on the Maxwell–Garnett (MG) approximation. This is because the MG model assumes that each ribbon is polarized only by the external field (illumination). However, the polarization of each ribbon in the grating also depends on the fields generated by the adjacent ribbons, implying that the grating needs to be treated as a spatially dispersive and thus nonlocal medium when the inter-ribbon coupling is strong and dependent on the polariton momentum *k.* Nonlocal modeling of polariton-coupled nanoribbons has recently been demonstrated with densely packed two-dimensional (2D) graphene nanoribbon arrays^8,9^. To that end, the nonlocal correction parameter $\sigma _{\mathrm{c}} = - i\frac{{2\omega \varepsilon _0L}}{\pi }\ln \left[ {\csc \left( {\frac{\pi }{2}\xi } \right)} \right]$ was introduced to calculate the effective conductivity of the graphene ribbon array according to $\sigma _{{\mathrm{eff}},x} = \left[ {(1 - \xi )/\sigma _{\mathrm{g}} + \xi /\sigma _{\mathrm{c}}} \right]^{ - 1}$ and $\sigma _{{\mathrm{eff}},y} = \left( {1 - \xi } \right){\upsigma}_{\mathrm{g}}$, with *σ*_g_ being the graphene conductivity. We employ this model to obtain the corrected permittivity model of our hBN metasurface of finite thickness *h*. To that end, we use the relation $\sigma \cong - i\omega h\varepsilon _0\varepsilon$ to obtain the nonlocal correction parameter $\varepsilon _{\mathrm{c}} = \frac{{2L}}{{\pi h}}\ln \left[ {\csc \left( {\frac{\pi }{2}\xi } \right)} \right]$ that is used in Eq. 1. Treating the hBN metasurface as a 2D conductivity sheet is justified since the hBN grating thickness *h* and the grating period *L* are much smaller than the polariton wavelength (i.e., *λ*_p_ » *h*, *L*) (ref. ^6,31^). Note that in our previous work^5^, we used the standard MG permittivity model (i.e. without the parameter *ε*_c_) to describe in-plane hyperbolic phonon polariton on the same hBN metasurface. This is possible as the polaritonic coupling of the hBN ribbons is negligible within the in-plane hyperbolic frequency range.

The effective permittivity *ε*_eff,*x*_ calculated according to Eq. 1 (red line in Fig. 1b) reveals the emergence of a new TO phonon frequency at *ω*_STO_ = 1478 cm^−1^, perpendicular to the ribbons. It results from the strong collective near-field coupling of the dipolar PhP resonance of the individual hBN ribbons (Supplementary Figs. 1–3), analog to the TO phonon in polar crystals. For this reason, we name this collective mode a synthetic TO phonon (STO)^32^. Note that the standard MG effective medium model also predicts the existence of the STO resonance (dashed gray line in Fig. 1b). However, it is shifted by about 50 cm^−1^ to higher frequencies because polaritonic near-field coupling and nonlocal effects induced by the grating geometry are not considered. As shown below, the STO predicted by our modified effective medium model (Eqs. 1 to 3) is in excellent agreement with both numerical and experimental results (see Fig. 1c, Fig. 3 and Supplementary Figs. 1 and 4).

### Numerical verification of anisotropic polaritons in the hBN metasurface

As a result of the STO, the photonic local density of states (PLDOS) on the metasurface differs dramatically from the one of natural hBN flakes, as confirmed by the calculated PLDOS spectra shown in Fig. 1c. In the simulations a point dipole source is placed at the height of 200 nm above the surface (see Methods). The PLDOS spectrum of the hBN layer of *h* = 20 nm thickness exhibits a strong peak around *ω* =1450 cm^−1^ (dashed gray line), due to the excitation of a fundamental PhP “waveguide” mode in the hBN slab^22,26^. In contrast, the metasurface (modeled as a *h* = 20 nm thick layer of an effective medium according to Eqs. 1–3) exhibits two PLDOS peaks located on either side of the STO (at 1430 and 1480 cm^−1^, blue line). These two peaks are verified by a full-wave numerical simulation using a real 20-nm-thick grating structure (red line) and indicate that two distinct PhP modes are excited on the metasurface (Supplementary Fig. 3). This result further corroborates the validity of the modified effective medium theory described by Eqs. 1–3 (in contrast to the standard MG theory, which does not account for the strong near-field coupling of polariton modes and fails in quantitative prediction of the STO frequency).

Below the STO frequency, the dipole excites PhPs possessing an in-plane hyperbolic dispersion^5^ (because Re(*ε*_eff,*x*_) > 0 and Re(*ε*_eff,*y*_) < 0), which are formed by near-field coupling of the waveguide polaritons propagating along individual nanoribbons (note that this coupling is weak and thus yields only a positive value for *ε*_eff,*x*_). The propagation of these in-plane hyperbolic PhPs (HPhPs) is highly anisotropic along the metasurface, exhibiting the typical ray pattern of hyperbolic polaritons (Fig. 1d). Fourier transform (FT) of the near-field distribution *E*_*z*_ indeed yields a hyperbolic IFC describing the polariton momentum **k** (Fig. 1e) in momentum space at fixed frequency.

Above the STO frequency, the dipole-excited PhPs have extremely elliptical in-plane dispersion, owing to *ε*_eff,*x*_ and *ε*_eff,*y*_ being negative but with largely different absolute values. The elliptical PhPs (EPhPs) are due to the strong collective near-field coupling (yielding a negative *ε*_eff,*x*_) of individual nanoresonators (i.e. the nanoribbons exhibiting Fabry–Pérot polariton resonances, see our verifications in Fig. 3 and Supplementary Fig. 7), which are visualized by the simulation shown in Fig. 1f, where the dipole source launches a collimated polariton beam with lateral confinement of about 150 nm (∼*λ*/45). The FT of the near-field distribution confirms the polaritons´ extreme elliptical IFC in **k**-space (Fig. 1g; note that the IFC is not a perfectly closed ellipse owing to polariton damping by intrinsic material losses).

Although it could be expected that EPhPs near the STO may suffer from the large imaginary part of *ε*_eff,*x*_ (Supplementary Fig. 5), their absolute propagation length is comparable to the one of HPhPs (Fig. 1d and Supplementary Fig. 6). This can be explained by the large negative real part of *ε*_eff,*x*_ near the STO, which actually reduces the field confinement inside the material, repelling the fields and hence reducing the absorption^17^. By increasing the frequency, the EPhPs become more confined and decay faster (Fig. 1h, more simulations shown in Supplementary Fig. 4), exhibiting a weaker ellipticity (Fig. 1i) owing to the decreasing figure of merit |Re(*ε*_eff,*x*_)|/|Re(*ε*_eff,*y*_)|. The highly collimated EPhP modes described in Fig. 1f, h are also known as canalization modes^17,21^. They have been explored in bulk metamaterials^21,33^ or two-dimensional metasurfaces^17^ for various applications, including hyperlensing^9,17,34^ and subwavelength focusing^33^. Theory also has been predicting the canalization of plasmon polaritons on metallic metasurfaces (graphene^17^, black phosphorus^13^, or metals^7^) at optical (visible and infrared) frequencies, which, however, has not been experimentally demonstrated. In our work we do not only theoretically predict the canalization of low-loss deeply-confined phonon polaritons but also demonstrate them experimentally via infrared nanoimaging.

### Polariton-interferometric nanoimaging of HPhPs and EPhPs

**Fig. 2:**
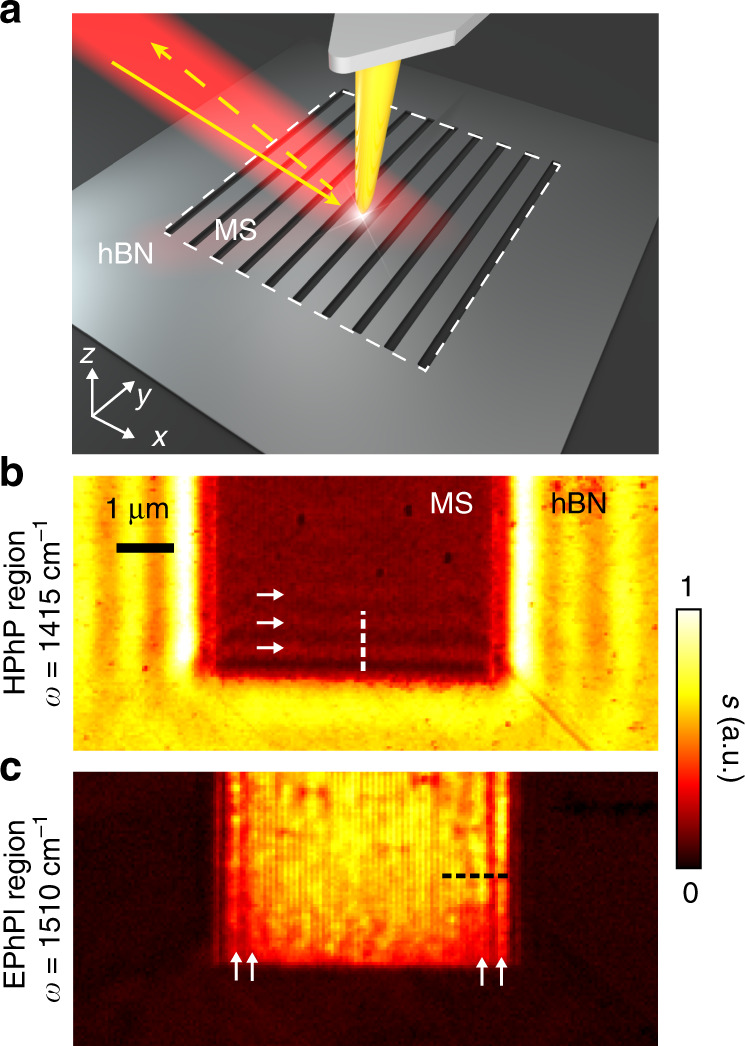
Near-field imaging of polariton evolution in a hBN metasurface. **a** Schematic of the near-field nanoimaging experiment. **b**, **c** Near-field images measured at two different frequencies, *ω* = 1415 cm^−1^ (HPhP region) and *ω* = 1510 cm^−1^ (EPhP region). White arrows indicate the polariton fringes observed on the metasurface.

To image the canalization of EPhP modes, we fabricated a hBN-metasurface (*w* = 75 nm and *g* = 25 nm) on a 20-nm-thick flake of monoisotopic low-loss hBN^5,28,35,36^ (schematics are shown in Fig. 2a; for details see Methods). We first performed polariton-interferometric nanoimaging on the metasurface with a scattering-type scanning near-field optical microscope (s-SNOM)^37,38^. The metallized tip of an atomic force microscope (AFM) is illuminated by a p-polarized infrared laser beam, operating as an infrared nanoantenna that concentrates the incident field at its sharp apex, yielding a nanoscale near-field spot for launching the polaritons. The polaritons propagate away and are reflected at the boundaries of the metasurface. They propagate back and interfere with the polariton field below the tip, forming interference fringes (with spacing equals to half the polariton wavelength, *λ*_p_/2), which are visualized by recording the tip-scattered field as a function of tip position^37,38^.

Figure 2b, c present polariton-interferometry images (amplitude signals, *s*) taken at frequencies within the HPhP and EPhP regions, respectively at *ω* = 1415 and *ω* = 1510 cm^−1^. On the grating area, we observe only horizontal fringes in the HPhP spectral range (Fig. 2b), and only vertical fringes in the EPhP spectral range (Fig. 2c). The two distinct fringe orientations reveal the different propagation directions of the polaritons, as predicted in our simulations shown in Fig. 1d, h, and h. They provide a first experimental indication for the existence of EPhPs above STO.

### Hyperspectral nanoimaging of polariton evolution in the hBN metasurface

**Fig. 3:**
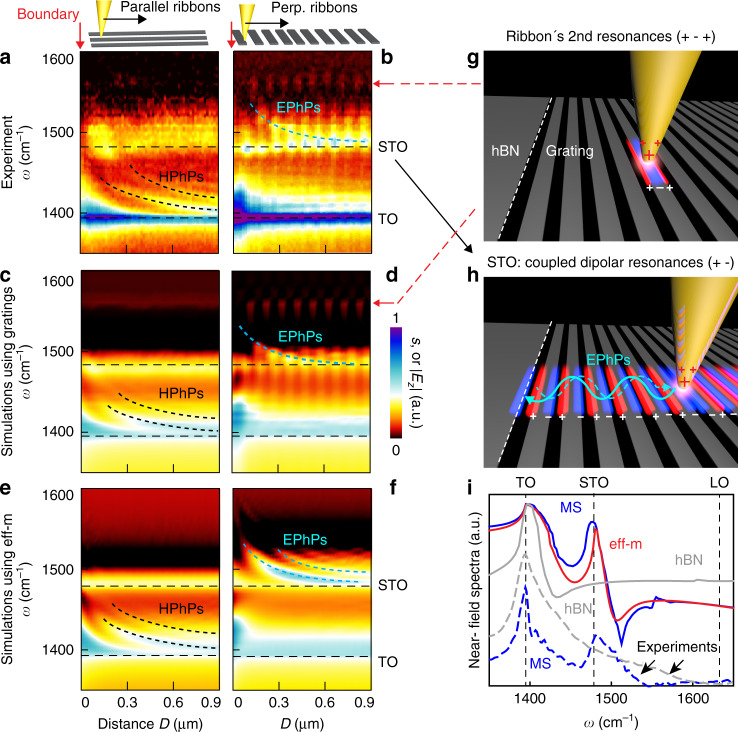
Hyperspectral nanoimaging of polariton evolution in a hBN metasurface. Near-field spectroscopic line scans taken parallel (**a**) and perpendicular (**b**) to the ribbons, as indicated in the top schematics. **c**–**f** Simulated line scans based on two different models, **c**, **d** for real grating structures, **e**, **f** for the effective medium (eff-m). **g**, **h** Schematics of tip-excitation of the second- and first-order dipolar PhP resonances of the hBN ribbons, respectively. **i** Dashed lines show experimental near-field spectra taken from the metasurface (on a gap of the grating, dashed blue line) and on the unstructured hBN (dashed gray line). Solid lines show simulated near-field spectra of the grating (blue line), of eff-m (red line) and of the unstructured hBN (gray line).

To explore the frequency range and dispersion of the two types of polaritons, we recorded near-field spectroscopic line scans (along the lines marked in Fig. 2b, c). In the line scan parallel to the ribbons (Fig. 3a), we observe a horizontal feature around *ω* = 1400 cm^−1^, matching well the TO phonon of hBN (see Fig. 3i for a comparison of experimental and simulated near-field spectra). Above the TO, we observe a series of fringes (indicated by dashed black curves), whose spacing is reducing with increasing frequencies. They reveal the in-plane HPhPs propagating parallel to the ribbons, whose wavelength is shrinking at higher frequencies. Around *ω* = 1500 cm^−1^ we observe a broad horizontal (non-dispersive) feature that fits well the STO (see Fig. 3i).

In the line scan perpendicular to the grating (Fig. 3b), we again observe the horizontal features corresponding to the TO phonon and the STO, respectively (see also Supplementary Fig. 7). In the whole spectral region between TO and STO (the HPhP range) we do not observe fringes, indicating the absence of polariton propagation perpendicular to the grating. More interestingly, we see an interference fringe (marked by a blue dashed curve) above STO. Its distance to the boundary decreases with increasing frequency, corroborating that the fringes in Fig. 2c indeed reveal a polariton propagating perpendicular to the grating. We note that the fringe intensity is modulated by the ribbons. Inside the gaps between the ribbons, the fringe intensity is higher, as here the tip more efficiently launches the polariton propagating perpendicular to the ribbons. The tip-launched polariton is reflected at the boundary, giving rise to the observed fringe (illustration in Fig. 3h). This propagating polariton mode is actually caused by the polaritonic near-field coupling of neighboring ribbons, similar to energy transport in plasmonic particle chains^39^. We further observe a horizontal series of bright spots at *ω* = 1570 cm^−1^. However, this feature is not accompanied by interference fringes at higher frequencies, indicating a purely localized mode. A zoom-in image and analysis (Supplementary Fig. 7) indeed show that the bright spots correspond to a localized second-order transverse polariton resonance of the ribbons, as illustrated in Fig. 3g.

Numerical simulations of the spectroscopic line scans (Fig. 3c, d; a dipole source was scanned above the grating and the field below the dipole was recorded, see Methods) confirm our experimental results, particularly the interference fringe (dashed blue curve) above the STO and the localized ribbon resonance around *ω* = 1570 cm^−1^ (marked by red arrow). We repeated the simulations for a metasurface described by the nonlocal effective medium theory described by Eqs. 1 to 3 (Fig. 3e, f), reproducing well the results of Fig. 3c, d. However, the signal modulation introduced by the grating is absent (due to spatial homogenization of the metasurface), thus yielding a clearer map. The good agreement of the different simulations confirms the validity of our nonlocal effective medium model, which is particularly important to properly capture the properties of the canalization polaritons near the topological transition.

Altogether, Fig. 3a, b experimentally verify two spectral regions (separated by the STO) within the hBN Reststrahlen band, in which two different types of polaritons exist. Their different propagation directions provide experimental evidence that the IFC of the polariton momentum undergoes a topological transition across the STO. To demonstrate tunability of the STO resonance experimentally (theoretical calculations shown in Supplementary Fig. 2), we show in the Supplementary Fig. 8 that the STO can be tuned from 1480 cm^−1^ (for the metasurface shown in Fig. 3 with ribbon width *w* = 75 nm and gap size *g* = 25 nm) to 1460 cm^−1^ by fabricating a metasurface with a different filling ratio (*w* = 220 nm and *g* = 40 nm).

### Real-space imaging of canalization polariton modes

**Fig. 4:**
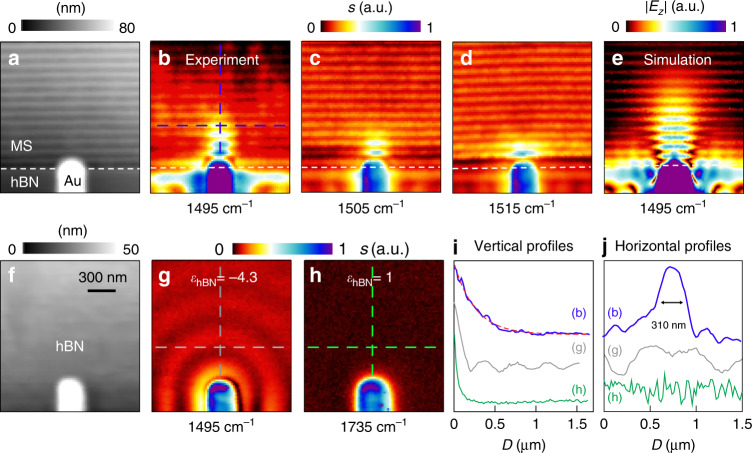
Real-space imaging of polariton canalization in a hBN metasurface. **a** Topography of the sample. **b**–**d** Experimental near-field distribution of antenna-launched elliptical polaritons on the metasurface at three different frequencies. White dashed lines mark the boundary of the metasurface. **e** Simulated near-field distribution of the antenna-launched canalization EPhPs on the metasurface. **f**, **g**, **h** Topography and experimental near-field images (at two different frequencies) for the case of an antenna located on an unpatterned area of the same hBN flake. **i** Blue line shows the near-field profile taken from the image shown in the panel (**b**) along the vertical dashed blue line, which can be fitted by an exponential decay (dashed red line). Gray line shows the near-field profile taken from **g** along the vertical dashed gray line. Green line shows the near-field profile taken from **h** along the vertical dashed green line. **j** Horizontal near-field line profiles taken from **b**, **g**, and **h** along the horizontal dashed lines, respectively.

To experimentally visualize the canalization mode near the STO, predicted in Fig. 1f, h, we imaged the polariton emitted from an infrared antenna (a gold rod) on another metasurface (fabricated together with the one in Fig. 2 on the same flake, topography in Fig. 4a). The antenna concentrates the mid-infrared illumination to nanoscale spots at its antenna extremities, acting as a nanoscale source for launching the polaritons. Figure 4b–d presents the experimental images of the antenna-launched polaritons propagating and decaying along the metasurface (see also Supplementary Fig. 9). In the grating area, periodic horizontal bright lines are observed. As explained in Fig. 3, they correspond to the strong near-fields inside the gaps, because of enhanced tip-polariton coupling. More importantly, we indeed observe the deep-subwavelength canalization EPhP — a collimated polariton beam (with a lateral confinement of 310 nm, ∼λ/22, see Fig. 4j; see also the FT results in Supplementary Fig. 10) emitted from the antenna extremity. At *ω* = 1495 cm^−1^ it is collimated over at least five ribbons (Fig. 4b). At higher frequencies, the polaritons are more confined and thus decay faster (Fig. 4c, d), but they still extend farther than the antenna fields decaying on a dielectric substrate (*ε*_hBN_ ≈ 1 at *ω* = 1735 cm^−1^, Fig. 4h). In a control experiment, we imaged the polaritons launched by the antenna on the un-patterned hBN (Fig. 4g, topography in Fig. 4f), showing radial propagation along the hBN, in striking contrast to the canalization modes (Fig. 4b–d).

We numerically verified the antenna-launched canalization polaritons by simulating the electromagnetic near-field distribution around the antenna on the grating structure (Fig. 4e). On the other hand, the simulated canalization mode propagates longer than the experimental one, which can be explained by stronger damping in the experiment caused by fabrication uncertainties and material damage from etching (see discussion section below).

## Discussion

An intriguing result of our experiments (Fig. 4b–d) is the direct visualization of energy flow transported along a chain of coupled polaritonic nanoresonators^39^. More precisely, we use the antenna to locally illuminate the first ribbon. Energy flows directionally to the next ribbons owing to the polaritonic near-field coupling and the extreme in-plane anisotropy of the canalization mode, which avoids energy spreading in other directions. The electric-field decay length for this process is quantified to be about 220 nm (blue line in Fig. 4i, background subtracted, see Supplementary Fig. 11) by fitting the near-field profile (along the vertical dashed blue line in Fig. 4b) with an exponential decay (dashed red line in Fig. 4i). This value is much larger than the one of antenna fields on the bare dielectric substrate (green line in Fig. 4i, decay length < 50 nm). Our results therefore provide a direct real-space observation of energy flow through coupled infrared phonon-polaritonic nanoresonators separated by nanoscale air gaps, with important consequences for the development of infrared photonic and thermal devices based on near-field polaritonic coupling. These results also confirm the important role of strong coupling between neighboring resonators to achieve extreme anisotropy and canalization. The consequent nonlocality, well captured by our homogenized metasurface model, plays an important role in the physics demonstrated in this paper.

We finally discuss the lifetime of the canalization polaritons. According to simulations (Supplementary Fig. 6), the canalization polaritons on the metasurface exhibit a lifetime that is comparable to that of phonon polaritons in hBN slabs that have the same thickness as the metasurface. In the experiment, however, the measured propagation length is about 2.5 times shorter. We explain this finding by additional polariton damping caused by polariton scattering and absorption at inhomogeneities and eventually material damage at the ribbon edges induced by etching. The simulations also reveal that the polariton propagation lengths and lifetimes can be increased at least by a factor of 2 by removing the relatively lossy SiO_2_ substrate (by suspending the metasurface) or by replacing it by a low-loss substrate such as CaF_2_. It thus can be expected that improving the fabrication process and employing low-loss substrates can enhance the propagation length of the canalization polaritons in potential future applications.

STOs (strong collective coupling of the metasurface elements) and topological transitions may also be envisioned in other types of metasurfaces, for instance based on strongly coupled graphene (or black phosphorus) nanoresonators^8,9^, which may lead to electrically-tunable collective resonances and canalization polaritons for sensing and thermal emission applications at infrared and THz frequencies. The demonstrated deep-subwavelength canalization polaritons hold promise for many exciting applications, including in-plane hyperlensing^8,9^, on-chip collimated polariton emitting, waveguiding, and focusing^8,9,17^.

## Methods

### Nanoimaging

We used a commercial s-SNOM system (from Neaspec GmbH) based on an atomic force microscope (AFM). The Pt-coated AFM tip (oscillating vertically at a frequency Ω ≈ 270 kHz) was illuminated by light from a wavelength-tunable continuous-wave quantum cascade laser. The backscattered light was collected with a pseudo-heterodyne interferometer^40^. To suppress background contribution in the tip-scattered field, the interferometric detector signal was demodulated at a higher harmonic *n*Ω(*n* ≥ 2), yielding near-field amplitude *s*_*n*_ and phase *φ*_*n*_ images. Figures 2 and 4 show amplitude *s*_3_ images.

### Spatio-spectral near-field observation

For spatio-spectral observation shown in Fig. 3, the s-SNOM tip and sample were illuminated with a broadband mid-infrared laser. The tip-scattered signal was analyzed with an asymmetric Fourier transform spectrometer (based on a Michelson interferometer), in which tip and sample were located in one of the interferometer arms^25,26^. An interferogram was measured by recording the demodulated detector signal (the harmonic 3Ω for background suppression) as a function of the position of the reference mirror, at a fixed tip position. Subsequent Fourier transform of the recorded interferogram yields a complex-valued near-field point spectrum^25,26^. We scanned the tip parallel or perpendicular to the hBN ribbons, respectively. At each tip position, we recorded a complex-valued near-field point spectrum. By plotting the recorded near-field amplitude *s*_3_ as a function of the tip position and the operation frequency, we obtained the images shown in Fig. 3a, b.

### Sample preparation

For experiments we used isotopically (^10^B) enriched hBN (details of the growing process can be found in refs. ^35,36^), which exhibits ultra-low-loss phonon polaritons^28^. We fabricated infrared metasurfaces by the etching process reported in ref. ^5^.

### Numerical simulations

We used a finite-element-method based software (COMSOL Multiphysics) for simulations. In the simulations, the permittivity of the isotopically enriched hBN was taken from ref. ^5^. Simulations of the real grating metasurface (referred to as grating and/or MS) consider the real three-dimensional geometry (given by *w*, *g*, *L,* and *h*) and hBN permittivity. Simulations of the homogenized metasurface (referred as to eff-m) consider a homogeneous slab of thickness *h=*20 nm with effective permittivities *ε*_eff,*x*_, *ε*_eff,*y,*_ and *ε*_eff,*z*_ described in Eqs. 1–3. Further details of the simulations are provided in the Supplementary Note 1.

## Supplementary information

Supplementary information

## Data Availability

The data that support the findings of this study are available from the corresponding author on reasonable request.

## Code availability

All codes used to evaluate the data are available from the corresponding author upon request.

## References

[1] Kildishev AV, Boltasseva A, Shalaev VM (2013). Planar photonics with metasurfaces. Science.

[2] Basov, D. N., Fogler, M. M. & García De Abajo, F. J. Polaritons in van der Waals materials. *Science* **354**, aag1992 (2016). 10.1126/science.aag1992 27738142

[3] Low T (2017). Polaritons in layered two-dimensional materials. Nat. Mater..

[4] High AA (2015). Visible-frequency hyperbolic metasurface. Nature.

[5] Li P (2018). Infrared hyperbolic metasurface based on nanostructured van der Waals materials. Science.

[6] Ma W (2018). In-plane anisotropic and ultra-low-loss polaritons in a natural van der Waals crystal. Nature.

[7] Yermakov OY (2015). Hybrid waves localized at hyperbolic metasurfaces. Phys. Rev. B.

[8] Gomez-Diaz JS, Tymchenko M, Alù A (2015). Hyperbolic Plasmons and Topological Transitions over Uniaxial Metasurfaces. Phys. Rev. Lett..

[9] Gomez-Diaz JS, Alù A (2016). Flatland Optics with Hyperbolic Metasurfaces. ACS Photonics.

[10] Poddubny A, Iorsh I, Belov P, Kivshar Y (2013). Hyperbolic metamaterials. Nat. Photonics.

[11] Liu, Y. & Zhang, X. Metasurfaces for manipulating surface plasmons. *Appl. Phys. Lett*. **103**, 141101 (2013).

[12] Yang Y (2017). Hyperbolic spoof plasmonic metasurfaces. NPG Asia Mater..

[13] Nemilentsau A, Low T, Hanson G (2016). Anisotropic 2D materials for tunable hyperbolic plasmonics. Phys. Rev. Lett..

[14] Hu D (2019). Tunable Modal Birefringence in a Low-Loss Van Der Waals Waveguide. Adv. Mater..

[15] Chaudhary K (2019). Engineering phonon polaritons in van der Waals heterostructures to enhance in-plane optical anisotropy. Sci. Adv..

[16] Folland TG, Caldwell JD (2018). Precise control of infrared polarization using crystal vibrations. Nature.

[17] Correas-Serrano D, Alù A, Gomez-Diaz JS (2017). Plasmon canalization and tunneling over anisotropic metasurfaces. Phys. Rev. B.

[18] Cortes CL, Jacob Z (2017). Super-Coulombic atom-atom interactions in hyperbolic media. Nat. Commun..

[19] Liu Z, Lee H, Xiong Y, Sun C, Zhang X (2007). Far-field optical hyperlens magnifying sub-diffraction-limited objects. Science.

[20] Krishnamoorthy HNS, Jacob Z, Narimanov E, Kretzschmar I, Menon VM (2012). Topological transitions in metamaterials. Science.

[21] Belov PA, Hao Y (2006). Subwavelength imaging at optical frequencies using a transmission device formed by a periodic layered metal-dielectric structure operating in the canalization regime. Phys. Rev. B.

[22] Dai S (2014). Tunable phonon polaritons in atomically thin van der Waals crystals of boron nitride. Science.

[23] Caldwell JD (2014). Sub-diffractional volume-confined polaritons in the natural hyperbolic material hexagonal boron nitride. Nat. Commun..

[24] Li P (2015). Hyperbolic phonon-polaritons in boron nitride for near-field optical imaging and focusing. Nat. Commun..

[25] Dai S (2015). Subdiffractional focusing and guiding of polaritonic rays in a natural hyperbolic material. Nat. Commun..

[26] Yoxall E (2015). Direct observation of ultraslow hyperbolic polariton propagation with negative phase velocity. Nat. Photonics.

[27] Ambrosio A (2017). Mechanical Detection and Imaging of Hyperbolic Phonon Polaritons in Hexagonal Boron Nitride. ACS Nano.

[28] Giles AJ (2018). Ultralow-loss polaritons in isotopically pure boron nitride. Nat. Mater..

[29] Joshua, D. Caldwell, Igor Aharonovich, Guillaume Cassabois, James H. Edgar, Bernard Gil, D. N. B. Photonics with hexagonal boron nitride. *Nat. Rev. Mater*. **4**, 552–567 (2019).

[30] Hu G, Shen J, Qiu C, Alù A, Dai S (2020). Phonon Polaritons and Hyperbolic Response in van der Waals Materials. Adv. Opt. Mater..

[31] Alfaro-Mozaz FJ (2019). Deeply subwavelength phonon-polaritonic crystal made of a van der Waals material. Nat. Commun..

[32] Lu Y (1999). Optical properties of an ionic-type phononic crystal. Science.

[33] Tuniz A (2013). Metamaterial fibres for subdiffraction imaging and focusing at terahertz frequencies over optically long distances. Nat. Commun..

[34] Forati E, Hanson GW, Yakovlev AB, Alù A (2014). Planar hyperlens based on a modulated graphene monolayer. Phys. Rev. B.

[35] Hoffman TB, Clubine B, Zhang Y, Snow K, Edgar JH (2014). Optimization of Ni-Cr flux growth for hexagonal boron nitride single crystals. J. Cryst. Growth.

[36] Liu S (2018). Single Crystal Growth of Millimeter-Sized Monoisotopic Hexagonal Boron Nitride. Chem. Mater..

[37] Chen J (2012). Optical nano-imaging of gate-tunable graphene plasmons. Nature.

[38] Fei Z (2012). Gate-tuning of graphene plasmons revealed by infrared nano-imaging. Nature.

[39] Maier SA (2003). Local detection of electromagnetic energy transport below the diffraction limit in metal nanoparticle plasmon waveguides. Nat. Mater..

[40] Ocelic N, Huber A, Hillenbrand R (2006). Pseudoheterodyne detection for background-free near-field spectroscopy. Appl. Phys. Lett..

