# Supplementary material for: Collective near-field coupling and nonlocal phenomena in infrared-phononic metasurfaces for nano-light canalization
Source: Nat Commun. 2020 Jul 21;11:3663. doi: 10.1038/s41467-020-17425-9 (PMC7374561; doi:10.1038/s41467-020-17425-9)
Supplement: Supplementary file 1 — Supplementary information [file 41467_2020_17425_MOESM1_ESM.pdf]

Supplementary Information

Collective near-field coupling and nonlocal phenomena in infrared-phononic metasurfaces for nano-light canalization

Li et al.

Supplementary Note 1. Simulation details

For simulations in Fig. 1, an electric dipole source at the height of 200 nm above the surface is used to excite the polaritons. The metasurface is modeled as either real grating nanostructures or an effective medium (see descriptions in the corresponding text). The near-field distribution is taken from the plane at the height of 50 nm above the surface. For calculating the PLDOS spectra shown in Fig.1c, we use the method introduced by supplementary ref. 1. Because of the *z*-axis orientation of the dipole source, we investigated the *z* component of the PLDOS that is proportional to the *z* component of the Green tensor ($\mathrm{Im}[\mathrm{G}(\boldsymbol{r},\boldsymbol{r},\omega)]$). The quantity $\mathrm{Im}[\mathrm{G}_{zz}(\boldsymbol{r},\boldsymbol{r},\omega)]$ is also proportional to the *z* component of electric field (the real part, $\mathrm{Re}[E_z]$) at the position of the dipole source. Thus, by normalizing the numerically simulated electric field $\mathrm{Re}[E_z]$ at the position of the dipole source to that obtained in vacuum, we obtain the PLDOS normalized by the PLDOS in vacuum as shown in Fig.1c of the main text.

For simulations in Fig. 3, we scanned the dipole (200 nm above the surface) over the metasurface (either the grating or the effective medium) along two different directions, respectively. The electric field (at the height of 50 nm above the surface) below the dipole is recorded as a function of the spatial position and the operation frequency, yielding the figures shown in Fig.3c to f.

For simulations in Fig. 4e, we used an Au antenna on the grating to excite the canalization mode. For modeling the grating metasurface, we used the ribbon width w = 60 nm and the gap size g = 40 nm according to the fabricated structures. We used a plane wave to illuminate the antenna and the metasurface. The near-field distribution is taken from the plane 50 nm above the surface. All simulations took into account the Si/SiO_2 (250 nm thick) substrate.

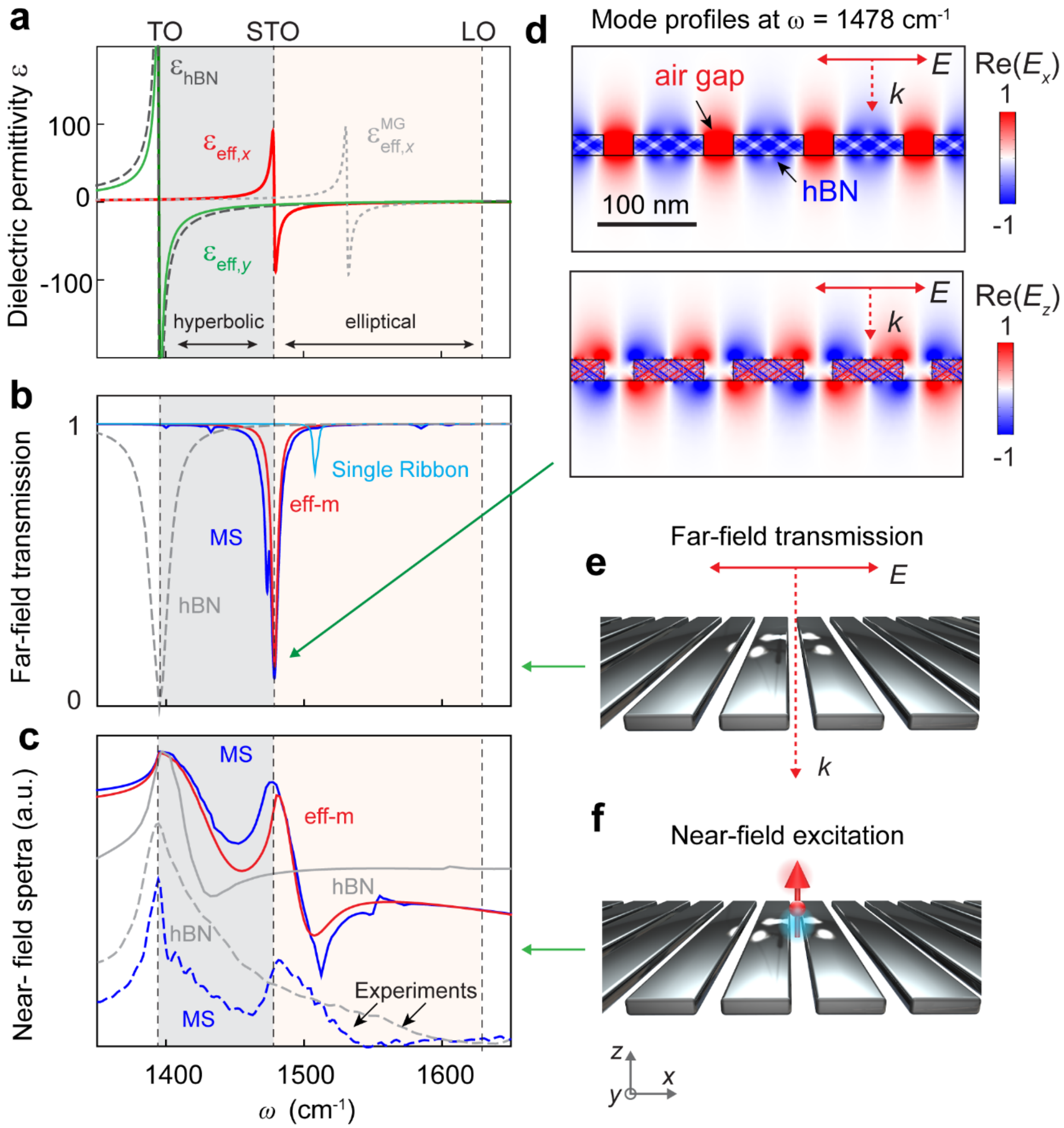

Supplementary Figure 1: Comparison of far-field and near-field spectra and effective permittivity. a Elements of the permittivity tensor as shown in Fig.1b of the main text. **b** Simulated far-field transmission spectra of the grating metasurface (blue), the metasurface modeled as an effective medium (eff-m, red), the un-patterned hBN (dashed grey) and single hBN nanoribbon (light blue). In these four cases, the layer thickness is the same as 20 nm. **c** Experiemental and simulated near-field spectra as presented in Fig.3i of the main text. **d** Simulated near-field distribution (the real parts of E_x and E_z) of the nanograting excited by a plane wave illumination at the frequency of the STO resonance ω =1478 cm^{-1}. **e** and **f** Schematics of the far-field transmission through the grating and near-field dipole excitation of the grating's resonance, respectively.

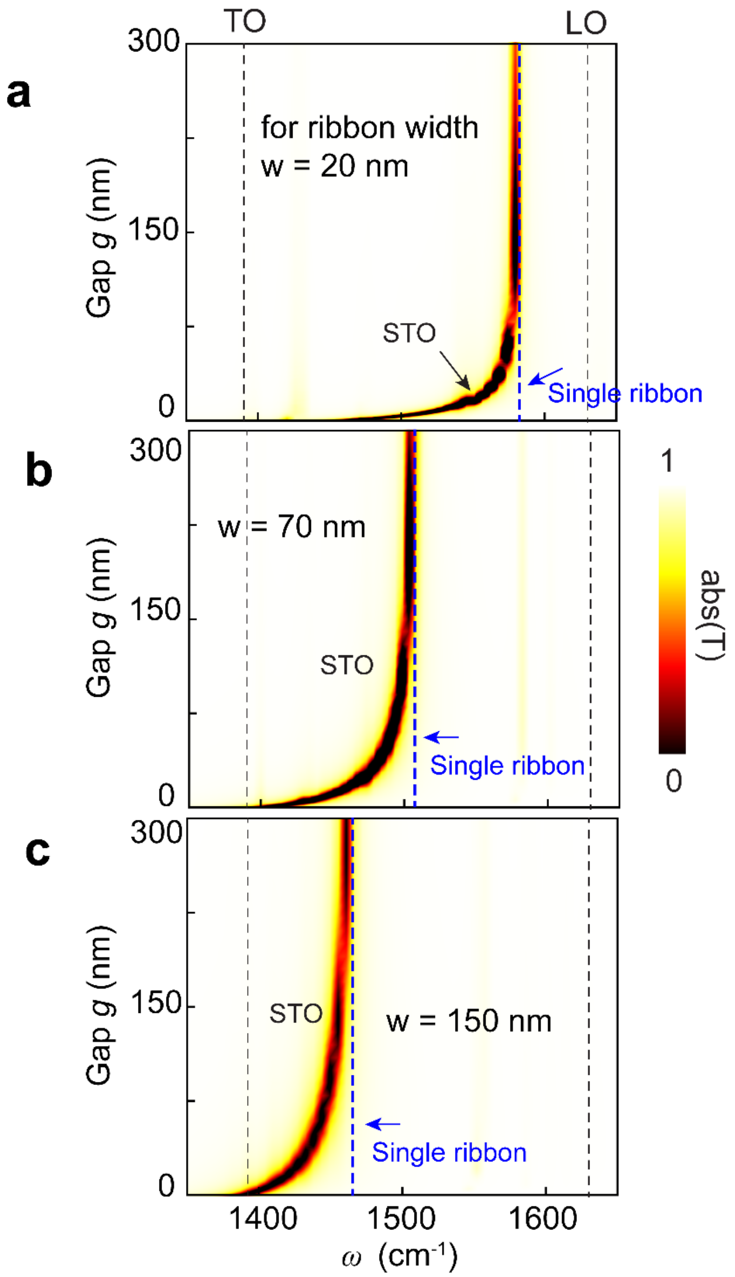

Supplementary Figure 2: Tuning the STO resonance with the gap size and the ribbon width. **a** to **c** Simulated far-field transmittance of the grating metasurface as a function of the air-gap size and the frequency, for (**a**) ribbon width *w*=20 nm, (**b**) *w*=70 nm, (**c**) *w*=150 nm. The vertical blue lines mark the resonance positions of single nanoribbons. This figure shows that the STO resonance of the metasurface can be well tuned by adjusting the sizes of both the gap and the ribbon width. For a fixed ribbon width, the resonance of single ribbon determines the upper limit of tuning the STO resonance with the gap size.

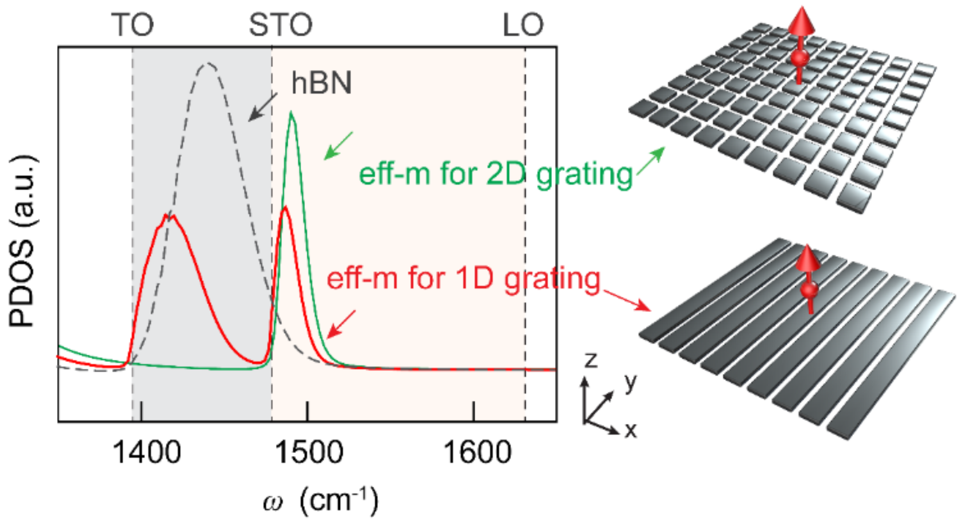

Supplementary Figure 3: Simulated PDOS spectra of 1D and 2D grating. The 1D grating exhibits two PDOS peaks that are due to the two different PhP modes (HPhPs and EPhPs) excited on the metasurface (modeled as an effective medium). In contrast, the 2D grating only exhibits a single PLDOS. This is because the 2D grating is the in-plane symmetrical, which thus supports an in-plane isotropic PhP mode. Therefore, this figure shows that the metasurface has great possibilities to engineer the polaritons propagating along the surface.

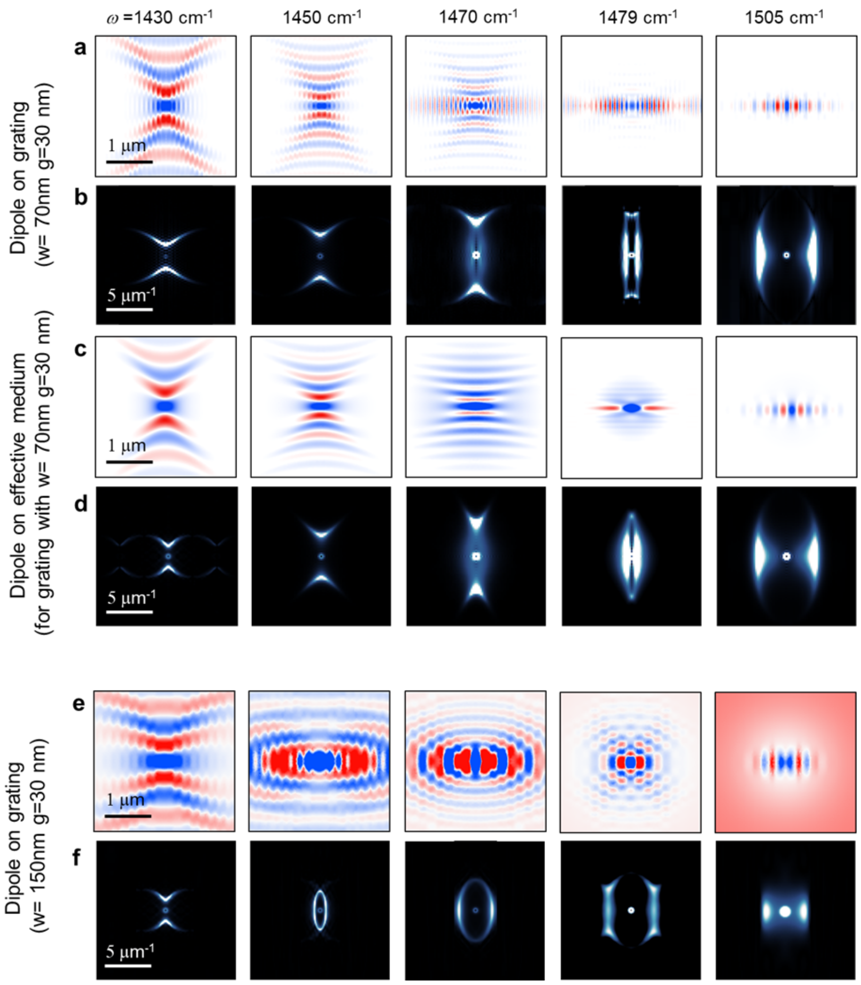

Supplementary Figure 4: Simulated near-field distribution and isofrequency curves of PhPs launched by a dipole source on different metasurfaces. (**a**) Metasurface modelled by grating w = 70 nm and g = 30 nm. (**c**) Metasurface modelled by an effective medium (obtained by Equation 1 in the main text). (**b**) and (**d**), Fourier transforms of the near-field distributions shown in panel (**a**) and (**c**), respectively. (**e**) Metasurface modelled by a grating with w = 150 nm and g = 30 nm. (**f**) the Fourier transform of the panel (**e**). Increasing the ribbon width redshifts the transition from the hyperbolic to the elliptical regime.

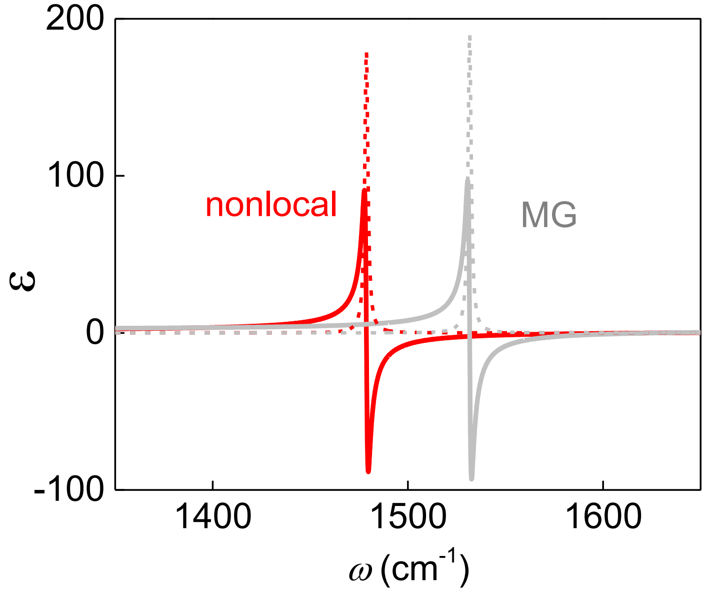

Supplementary Figure 5: Calculated effective permittivity $\varepsilon_{\mathrm{eff,x}}$ based on two distinct models. Nonlocal effective medium model, Equation 1 (red) and standard effective medium model based on Maxwell-Garnett (MG) approximation (grey). Solid lines, real parts; dashed lines, imaginary parts. Both models predict the existence of STO resonance. However, the one predicted by the MG model is shifted by at least 50 cm^{-1} to a wrong spectral position (as we experimentally corroborated in Fig.3 of the main text), because it does not consider the polaritonic coupling of the ribbons and the nonlocal effects induced by the structuring.

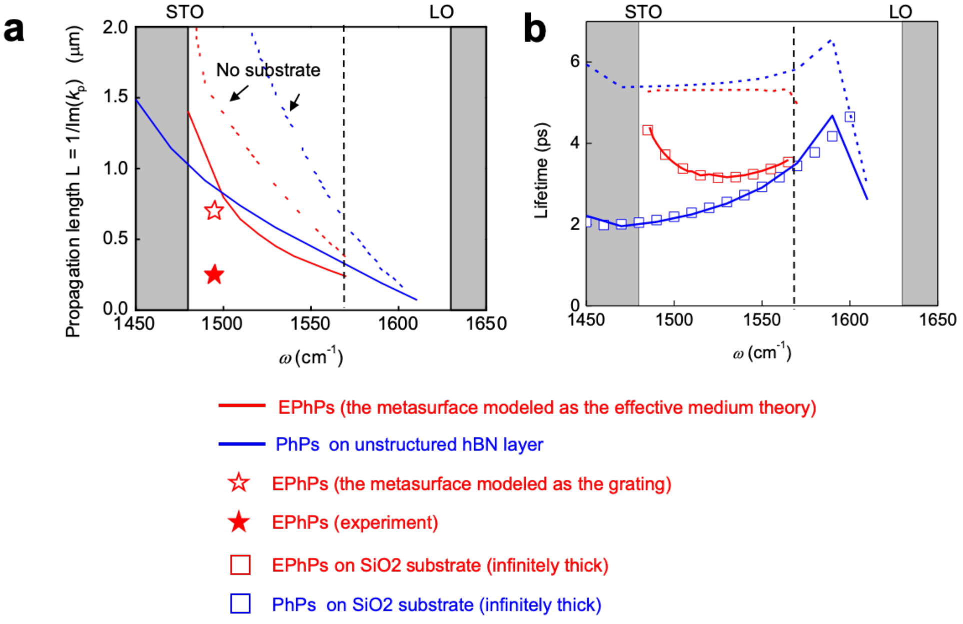

Supplementary Figure 6: Comparison of propagation length (a) and lifetime (b) of phonon-polariton on the metasurface and unstructured slab. We first calculate the propagation length and lifetime of EPhPs using the COMSOL software with the effective permittivity calculated from our nonlocal model (vertical dashed lines in the figures mark the frequency limit that our model is valid)**.** In the case with the SiO_2(250nm)/Si substrate, the EPhPs (red solid line) on the metasurface exhibit a similar propagation length and a longer lifetime compared to that of the phonon polaritons (blue solid line) in hBN slabs of corresponding thickness. Both the propagation length and the lifetime of EPhPs increase at frequencies close to the STO (the regime for canalization modes). This can be explained by the large negative real part of $\varepsilon_{eff,x}$ near the STO, which actually reduces the field confinement inside the material, repelling the fields and hence reducing the absorption. We also perform another simulation of placing the dipole to launch EPhPs on the nanograting (metasurface). We fit the propagation length of the dipole-launched canalization EPhP mode with an exponential decay model, which is plotted as an open symbol in the figure. The canalization EPhP on the nanograting exhibits a smaller propagation length (about 780 nm at 1495 cm^{-1}) compared to that of EPhPs simulated based on the effective medium model (about 1 μm at 1495 cm^{-1}). In the experiment, we observe that the canalization EPhP propagates only 220 nm (solid symbol), which is much smaller than the two theoretical values. This can be explained by higher damping in the experiment caused by fabrication uncertainties and material damage from etching. Note that the polariton lifetimes obtained on the SiO_2(250nm)/Si substrate are nearly identical with lifetimes obtained for semi-infinite SiO_2 substrate (open squares). This finding shows that the influence of the Si substrate is negligible, as the extension of the polariton field into the SiO_2 layer is much smaller than the thickness (250 nm) of the SiO_2 layer. We also perform the simulations for the cases without the substrate and observe that the polariton propagation length and lifetimes can be largely increased. Therefore, fabricating higher-quality metasurface on a lossless substrate can improve the propagation length of canalization EPhPs.

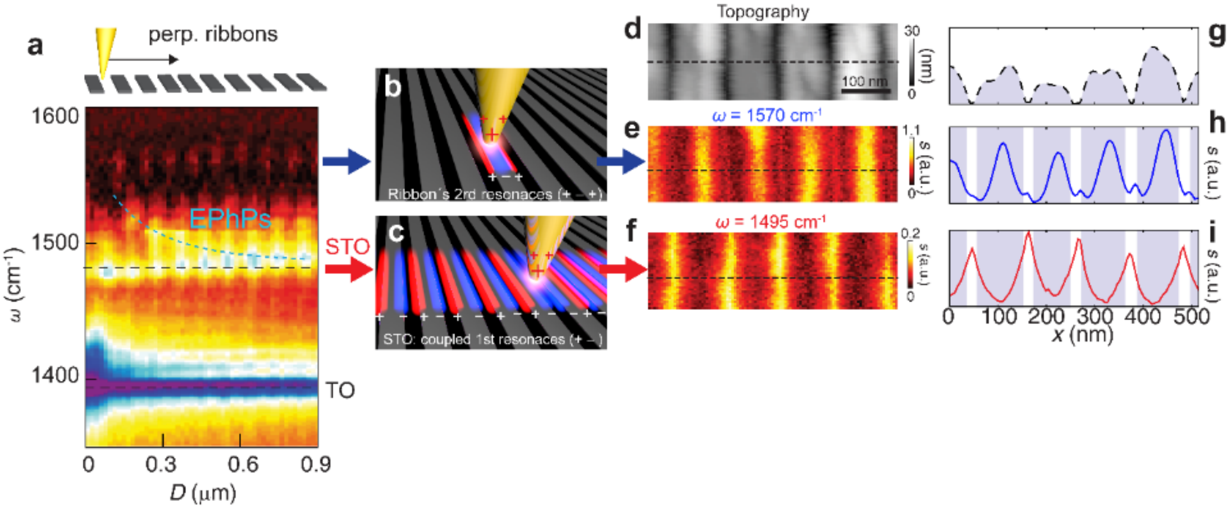

Supplementary Figure 7: Near-field imaging of the grating at the fundamental STO (coupled dipolar) resonance and at the second order (+ - +) resonance. **a** Spectroscopic near-field line scan perpendicular to the ribbons (shown in Fig.3b of the main text). **b** and **c** schematics of tip excitation of the STO resonance and the ribbons´ 2nd resonance (+ - +), respectively. **d** Topography of the grating. **g** Line profiles along the dashed line in (**d**). **e** and **f** Near-field images measured at the STO resonance (ω =1495 cm^{-1}) and the ribbons´ 2nd (+ - +) resonance (ω =1570 cm^{-1}). **h** and **i** Line profiles (averaged by 10 lines) along the dashed lines in (**e**) and (**f**). Clearly, the strong near-field signals are observed at the gaps for the STO resonance, while the strong near-field signals are found on the ribbons for the 2nd resonance (+ - +).

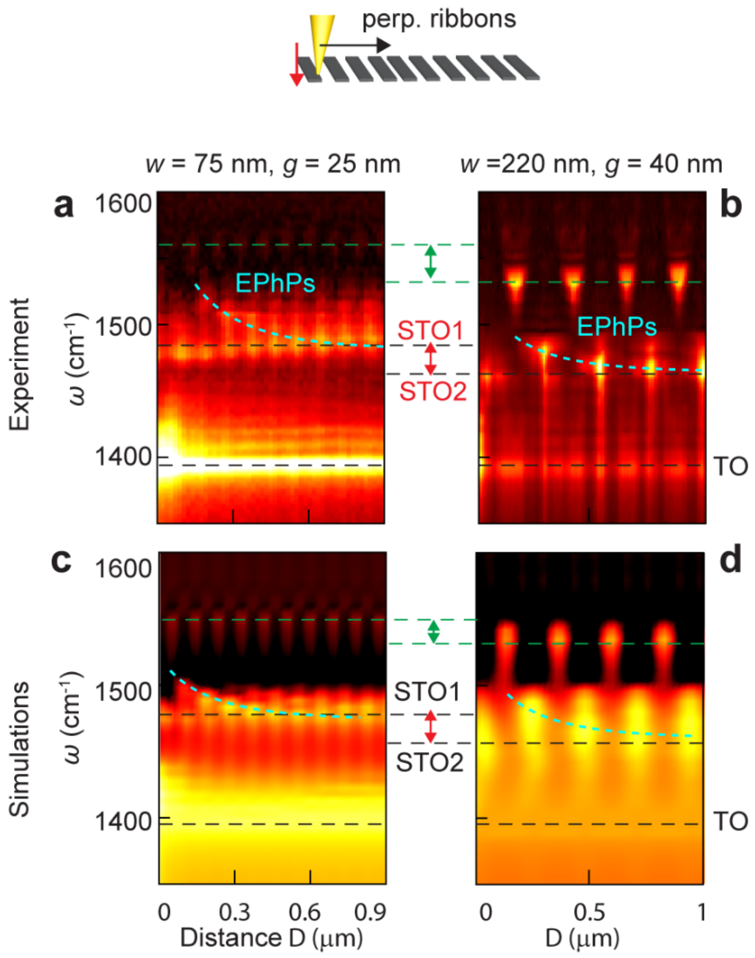

Supplementary Figure 8: STO resonance tuning via metasurface parameter. a, **b** Near-field spectroscopic line scans across two different metasurfaces, (a) the metasurface shown in Fig. 3 of the main text (w = 75 nm and g = 25 nm) and (b) a metasurface with w = 220nm and g = 40nm. The scans are taken perpendicular to the ribbons, as indicated in the schematics. The STO resonace shifts from 1480 cm^{-1} to 1460 cm^{-1} (indicated by red arrow between panel a and b). Further, the EPhP modes (indicated by dashed light-blue lines) and the 2nd-order polariton resonace (incidtated by dahsed green lines) of individual ribbons shift accordingly. **c**, **d**, Numerically simulated line scans, considering the full 3D structure of the metasurface. The simulations clealry verify the experimental observations of (a) and (b). The thickness of all metasurface samples has a similar thickness around 20 nm.

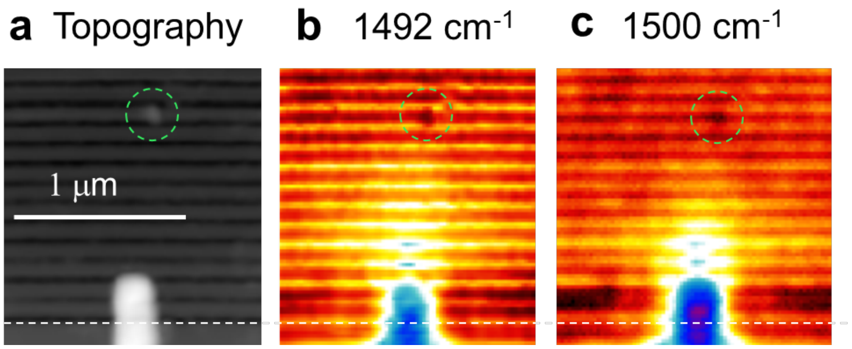

Supplementary Figure 9: Observation of EPhPs on another metasurface. a, Topography image. The metasurface has the same design parameters as the one shown in the main text. **b** and **c**, Near-field images of antenna-launched EPhPs on the metasurface taken at two different frequencies. Similar to Fig. 4 of the main text, we observe the directional propagation of EPhPs on the metasurface. The green cycles mark a dust particle on the metasurface. The dashed white lines mark the boundary of the metasurface.

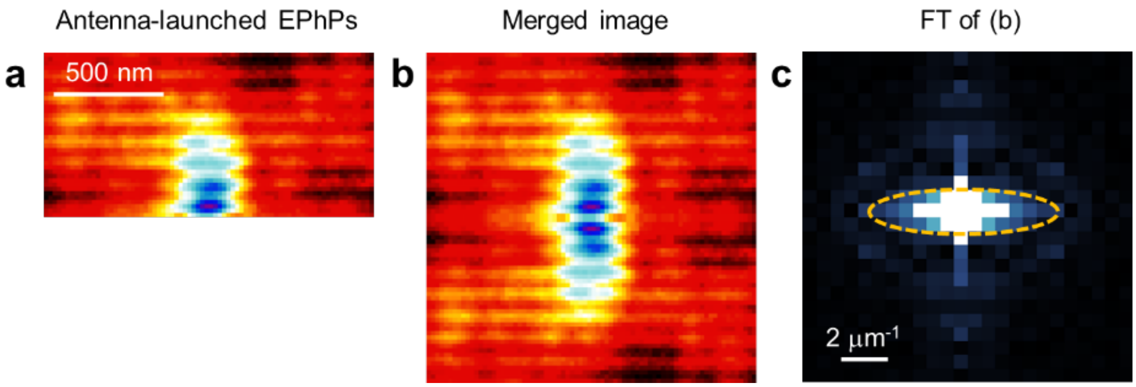

Supplementary Figure 10: Determination of experimental polariton isofrequency contours. a Near-field distribution of antenna-excited canalization EPhPs (at ω= 1495 cm^{-1}, as shown in Fig.4b of main text). **b** Image obtained by merging original and vertically flipped image of panel (a). This procedure increases the number of image pixels for the subsequent Fourier transform, in order to increase the number of pixels in the Fourier transform and thus its quality. **c** Fourier transforms (FT) of the image shown in panel (b) using the Gwyddion. In this image, we observe a compressed solid ellipse rather than the isofrequency contour (or surface) as theoretically predicted in Fig.1g, which could be because of the low resolution of the FT image. This compressed ellipse can be qualitatively described by the simulated isofrequency contour (dashed orange curve), revealing the canalization features of polaritons observed in real space. The discrepancy between the simulation and the experiment could be due to the size uncertainties of fabricated structures.

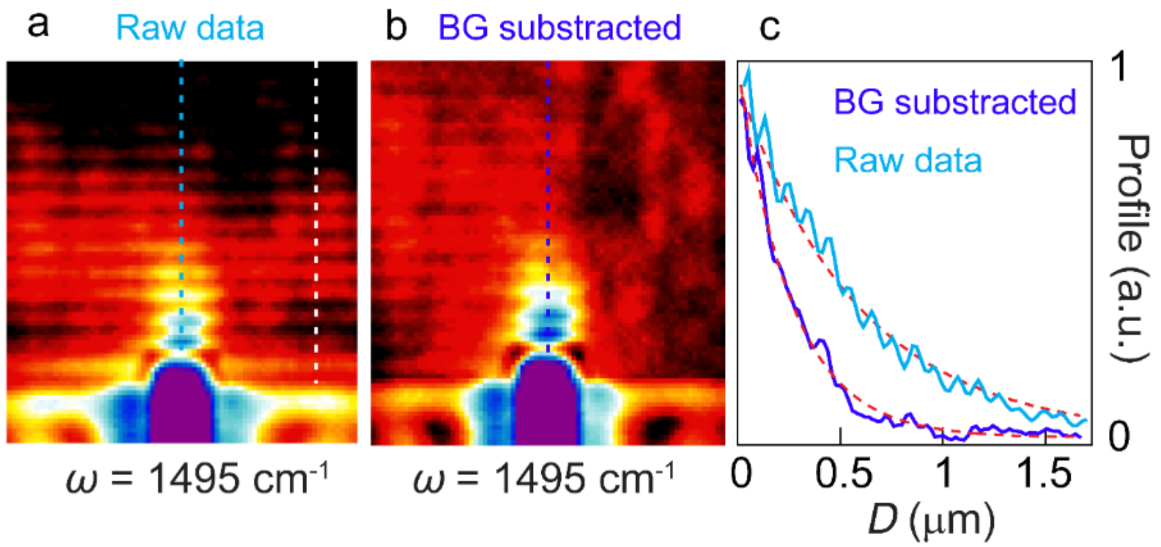

Supplementary Figure 11: Background（BG）subtraction for evaluating the decay length of the antenna-launched canalization PhPs. a Near-field image measured at ω=1495 cm^{-1}, as already shown in Fig.4b of the main text. **b** The resulting image of (**a**) after subtracting the background (averaged by 10 lines) along the white dashed line marked in (**a**). We performed the background subtraction by using a predefined functionality of an open-source software (Gwyddion)**.** **c** Solid lines, profiles along the dashed blue lines in (**a**) and (**b**). Dashed red lines, the fitting of the solid lines. For line profile of the raw data (**a**), we obtain the decay length about 590 nm. After the background subtraction, we obtain the decay length about 220 nm for the profile of (**b**).

Supplementary References

1. Peragut, F., Cerutti, L., Baranov, A., Hugonin, J. P., Taliercio, T., De Wilde, Y. & Greffet, J. J. Hyperbolic metamaterials and surface plasmon polaritons. *Optica*, **4**, 1409-1415 (2017).
